# N7‐Methylguanine‐Related Gene Signature Highlights *EIF4E* as a Novel Therapeutic Target in HER2‐Negative Breast Cancer

**DOI:** 10.1111/jcmm.70808

**Published:** 2025-08-21

**Authors:** Yangyang Cui, Yuhan Dai, Yiqin Xia, Wenxin Yu, Jiangdong Jin, Shui Wang, Hui Xie

**Affiliations:** ^1^ Department of Breast Surgery The First Hospital Affiliated Hospital With Nanjing Medical University Nanjing China; ^2^ Department of General Surgery Women's Hospital of Nanjing Medical University & Nanjing Women and Children's Healthcare Hospital Nanjing China; ^3^ Department of Breast Surgery Jiangsu Cancer Hospital & Jiangsu Institute of Cancer Research & Affiliated Cancer Hospital of Nanjing Medical University Nanjing China

**Keywords:** *EIF4E*, HER2‐negative breast cancer, N7‐methylguanosine, patient outcomes

## Abstract

Globally, breast cancer remains one of the most prevalent malignancies and a leading cause of cancer‐related death in women, with over 2.3 million new cases reported annually. Despite treatment advances, one breast cancer type in particular, HER2‐negative breast cancer, lacks precise therapeutic targets. Given the role of N7‐methylguanosine (m7G) in gene regulation and its links to cancer progression, we investigated m7G regulatory gene expression and prognostic potential in HER2‐negative breast cancer. We analysed publicly available breast cancer datasets (The Cancer Genome Atlas and the Gene Expression Omnibus (GEO)) to analyse the differential expression of 14 m7G‐regulatory genes. Clustering analysis, based on m7G patterns, categorised HER2‐negative patients into two subgroups. A prognostic model was established through LASSO and Cox regression; subsequently validated by survival analysis, and further supported by functional assays confirming gene function. Our model identified *CCNB1* and *EIF4E* as high‐risk genes, with *EIF4E* overexpression enhancing cell proliferation, migration and invasion. RNA‐sequencing (RNA‐seq) and pathway analyses showed that upregulated *EIF4E* activated Wnt signalling and extracellular matrix (ECM) components, processes required for tumour progression. High‐risk patients showed reduced immune cell infiltration and poorer survival outcomes. We highlight m7G regulatory gene potential, particularly *EIF4E*, as prognostic markers and therapeutic targets for HER2‐negative breast cancer. Targeting *EIF4E*‐related pathways could provide new therapeutic strategies to improve breast cancer patient outcomes.

## 1 Introduction

Globally, breast cancer is one of the most common malignancies and is a leading cause of cancer‐related death in women, with over 2.3 million new cases each year [1, 2]. Additionally, breast cancer is a highly heterogeneous tumour, with closely linked molecular characteristics, cellular composition and clinical prognosis. In recent decades, disease treatments have shifted from a ‘one‐size‐fits‐all’ model to a ‘personalised’ approach, significantly improving patient survival rates [3] and highlighting the importance of precision medicine in optimising patient outcomes [4].

Historically, breast cancer treatment has primarily relied on surgery, radiotherapy and systemic therapies such as chemotherapy and endocrine therapy. While these traditional therapies are effective for most patients, they have several limitations, such as non‐specific toxicity, drug resistance and limited efficacy in patients with advanced disease [5, 6, 7, 8]. With the advent of molecular biology and genomic technologies, the field has undergone a paradigm shift toward more targeted treatment strategies aimed at specific oncogenic drivers, thereby minimising off‐target effects and enhancing therapeutic outcomes [9, 10, 11].

The introduction of HER2‐targeted therapies, such as trastuzumab and pertuzumab, has revolutionised treatments for HER2‐positive breast cancer, providing significant survival benefits for patients [12, 13, 14]. While combining CDK4/6 inhibitors with endocrine therapy has extended progression‐free survival (PFS) in HR+/HER2‐patients [15, 16], precise therapeutic targets for HER2‐negative breast cancer are lacking.

Precision medicine, or personalised oncology, has emerged as a promising approach to overcome traditional therapy limitations. In using molecular technologies, such as next‐generation sequencing and other ‘omics’ methods, clinicians can customise treatment plans based on unique molecular and genetic alterations in a patient's tumour. The shift from a ‘one‐size‐fits‐all’ strategy to more tailored treatment plans for specific patients marks a major advancement in oncology [17, 18, 19]. In recent years, attention has also turned to epigenetics and post‐transcriptional modifications in cancer biology. RNA modifications, particularly N6‐methyladenosine (m6A) and N7‐methylguanosine (m7G), have been identified as key regulators of gene expression, impacting processes such as RNA stability, splicing and translation [20]. However, dysregulated modifications are associated with tumorigenesis and thus provide new insights into cancer pathophysiology and more novel therapeutic targets [21, 22, 23].

RNA modification is a crucial regulatory player in cellular processes, with over 150 types identified to date. Of these, m6A and m7G modifications have drawn considerable interest due to their regulatory roles at various RNA metabolism stages, from transcription to translation. In cancer, alterations in enzymes responsible for adding, removing or recognising these modifications (known as ‘writers’, ‘erasers’ and ‘readers’) can lead to abnormal gene expression patterns, promoting tumorigenesis and metastasis [24, 25, 26, 27]. Recent studies have highlighted the importance of m7G methylation in cancer progression, particularly in regulating mRNA stability and translation [28, 29]. The m7G modification commonly occurs at the 5′ end of eukaryotic mRNA, where it initiates translation [30]. Additionally, m7G is present in other non‐coding RNAs, including tRNA and rRNA, suggesting broader roles in cellular homeostasis [31, 32, 33]. In malignancies, dysregulated m7G‐modifying enzymes (such as METTL1 and WDR4) are associated with tumour progression and poor prognosis outcomes, positioning m7G as a potential biomarker and therapeutic target [34, 35, 36].

Given the lack of clear therapeutic targets for HER2‐negative breast cancer patients, and the significant role of m7G regulatory genes in disease onset and progression, we investigated m7G regulatory gene expression patterns in HER2‐negative breast cancer patients to identify new prognostic markers and therapeutic targets.

## 2 Materials and Methods

### 2.1 Data Collection and Analysis

Clinical and transcriptome data from 723 breast cancer samples (164 HER2‐positive and 559 HER2‐negative) were obtained from The Cancer Genome Atlas database (http://cancernome.nih.gov/) and used as the training set. Additionally, clinical and transcriptome data from 107 breast cancer samples (GSE58812) were collected from the Gene Expression Omnibus (GEO) database (https://www.ncbi.nlm.nih.gov/geo/) as an external validation set. Fourteen m7G‐related genes were selected from the previous literature and analysed. Differential gene expression between HER2‐negative and HER2‐positive samples was assessed using the ‘Limma’ R package.

### 2.2 Consensus Clustering and Subgrouping

To classify m7G methylation modification patterns, consensus unsupervised clustering was performed using the ‘ConsensusClusterPlus’ package in R. Clustering stability was evaluated using the cumulative distribution function (CDF) and principal component analysis (PCA) along with t‐distributed stochastic neighbour embedding (tSNE) for dimensionality reduction. The number of clusters (K) was set to two, resulting in two subgroups termed m7G Cluster 1 and Cluster 2.

### 2.3 Survival and Clinical Correlation Analysis

Patient survival analysis was conducted based on subgroups derived from consensus clustering. Survival differences between clusters were evaluated using Kaplan–Meier survival curves, and associations with clinical characteristics were examined using correlation analysis.

### 2.4 Construction of a Prognostic Model

A prognostic model was constructed based on m7G regulatory genes using univariate Cox regression analysis to identify genes that were significantly associated with HER2‐negative breast cancer prognosis outcomes. The significance cut‐off was *p* < 0.05. LASSO Cox regression was then used to further refine gene signatures, resulting in a two‐gene signature comprising *CCNB1* and *EIF4E*. A risk score was calculated for each patient using the formula: risk score = 0.0104873714482289 * *CCNB1* expression + 0.3036812520118 * *EIF4E* expression. Patients were divided into high‐ and low‐risk groups based on the median risk score.

### 2.5 Model Evaluation

The prognostic model was assessed using receiver operating characteristic (ROC) curves and area under the curve (AUC) analysis for 3‐, 5‐ and 8‐year survival predictions. Univariate and multivariate Cox regression analyses were performed to identify factors that were significantly associated with overall survival (OS), including lymph node metastasis, distant metastasis, stage and histological type.

### 2.6 Functional Enrichment Analysis

Differentially expressed m7G‐related genes between high‐ and low‐risk groups were identified using |log2 FC| > 1 and *p* < 0.05 cut‐off criteria. Gene Ontology (GO), Kyoto Encyclopedia of Genes and Genomes (KEGG) and Gene Set Enrichment Analysis (GSEA) were performed to explore biological processes and pathways associated with these genes.

### 2.7 Immunological Analysis

The CIBERSORT algorithm was used to estimate immune cell infiltration in high‐ and low‐risk groups. The TIMER algorithm was also used to assess immune infiltration levels, specifically B cells, and calculate immune scores and tumour immune dysfunction and exclusion (TIDE) immunogenicity scores [37, 38, 39].

### 2.8 Drug Sensitivity Analysis

The IC50 values of different small‐molecule drugs were obtained from the Genomics of Drug Sensitivity in Cancer (GDSC) database. Drug sensitivity was compared between high‐ and low‐risk groups to identify potential therapeutic agents for HER2‐negative breast cancer.

### 2.9 Tumour Mutation Burden (TMB) Analysis

The top 15 mutated genes in high‐ and low‐risk groups were identified, including *PIK3CA, TP53, TTN, CDH1* and others. TMB was calculated for each group, and TMB differences evaluated.

### 2.10 Cell Culture and Transfection

HER2‐negative breast cancer cell lines MCF‐7 and MDA‐MB‐231 were cultured in Dulbecco's Modified Eagle Medium plus 10% fetal bovine serum (Gibco, Australia) and 1% penicillin–streptomycin. Cells were grown at 37°C in 5% CO_2_. For transfection experiments, cells were seeded in 6‐well plates 24 h prior to transfection to achieve approximately 70% confluence. Cells were then transfected with either an EIF4E overexpression plasmid (pcDNA‐EIF4E) or an EIF4E‐specific small interfering RNA (siRNA) using Lipofectamine 3000 (Invitrogen, Carlsbad, CA, USA) according to the manufacturer's instructions.

EIF4E‐siRNA‐1 (Sense (5′‐3′): CAAAGAUAGUGAUUGGUUATT; Antisense (5′‐3′): UAACCAAUCACUAUCUUUGTT);

EIF4E‐siRNA‐2 (Sense (5′‐3′): GGCUAAUUACAUUGAACAATT; Antisense (5′‐3′): UUGUUCAAUGUAAUUAGCCTT).

### 2.11 Quantitative PCR (qPCR)

Total RNA was extracted from cells using Trizol reagent, after which cDNA was synthesised from 1 μg total RNA using HiScript IV All‐in‐One Ultra RT SuperMix for qPCR (Vazyme), with qPCR performed using SYBR qPCR Master Mix (Vazyme). For quality control, we performed all reactions in triplicate and confirmed single‐peak amplification for all primers through melting curve analysis. Cycle threshold (Ct) values were validated within the range of 15–35. *EIF4E* expression levels were normalised to *GAPDH* and analysed using the 2^−ΔΔCt^ method. Primers for *EIF4E* are forward primer: 5′‐GGTATTGAGCCTATGTGGGAAG‐3′, reverse primer: 5′‐TCGTCTCTGCTGTTTGTTCAATG‐3′ and *GAPDH*: forward primer: 5′‐GAACGGGAAGCTCACTGG‐3′, reverse primer: 5′‐GCCTGCTTCACCACCTTCT‐3′.

### 2.12 Colony Formation Assays

Cells transfected with *EIF4E*‐overexpression or *EIF4E*‐knockdown plasmids were seeded into 6‐well plates (500 cells/well) and cultured for 14 days. Colonies were fixed in methanol, stained in 0.1% crystal violet and counted.

### 2.13 Cell Proliferation Assays

Cell proliferation was assessed using a Cell Counting Kit‐8 (CCK‐8) (Dojindo, Japan). Transfected cells were seeded into 96‐well plates (2000 cells/well) and the absorbance at 450 nm was measured daily for 5 days.

### 2.14 Transwell Migration and Invasion Assays

Transwell chambers containing 8 μm pore inserts were used for migration and invasion assays. Cells were seeded in serum‐free medium in upper chambers, and lower chambers were filled with medium containing 20% FBS. After 48 h, migrated or invaded cells on membranes were fixed, stained in crystal violet and counted.

### 2.15 Wound Healing Assays

Cell motility was evaluated using wound healing assays. Cells were seeded in 6‐well plates and cultured until 90% confluent. A scratch was made in cell monolayers using a sterile 200 μL pipette tip, and images were taken at 0 and 24 h to assess wound closure.

### 2.16 RNA‐Sequencing (RNA‐Seq) and Bioinformatics Analysis

RNA was extracted from cells transfected with *EIF4E* plasmids and subjected to RNA‐seq. Differentially expressed genes were identified with a fold change > 2 and *p* < 0.05. KEGG and GO pathway enrichment analyses were performed to identify pathways associated with *EIF4E*‐regulated genes.

### 2.17 Statistical Analysis

Statistical analysis was conducted using SPSS version 20.0 (SPSS, Chicago, IL, USA) and GraphPad Prism 6 (GraphPad Software, CA, USA). Statistical significance was accepted at *p* < 0.05. All results were based on an average of at least three independent experiments.

## 3 Results

### 3.1 m7G Regulatory Gene Expression Patterns in HER2‐Negative/−Positive Breast Cancer Patients

**FIGURE 1:**
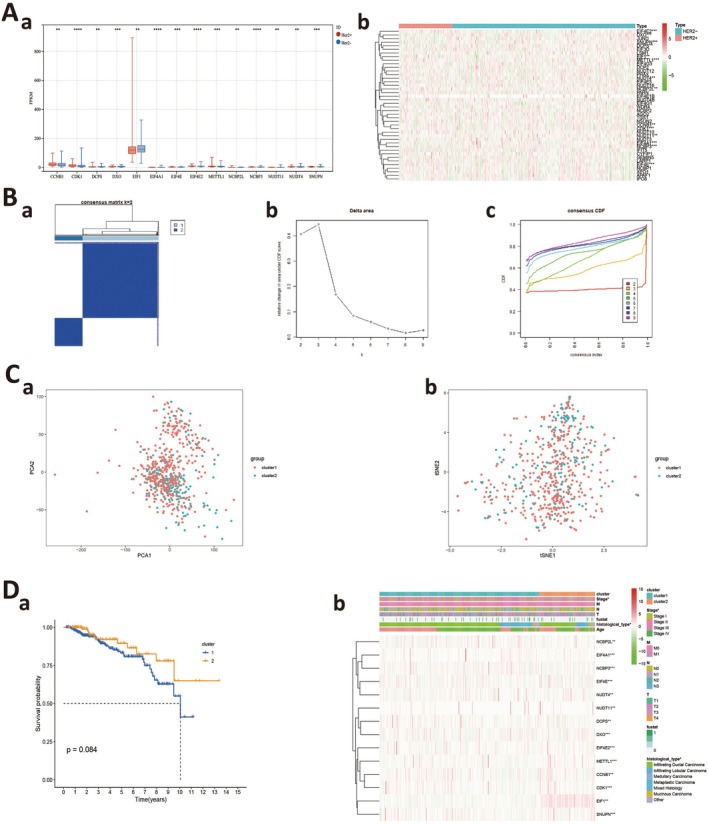
Expression and classification of m7G regulatory genes in HER2‐positive and HER2‐negative breast cancer patients. (A) (a, b) Expression of 14 m7G regulatory genes in HER2‐positive and HER2‐negative breast cancer patients. (B) (a–c) Using machine learning, unsupervised clustering of the 14 m7G genes effectively classifies HER2‐negative patients into two subgroups. (C) (a, b) Principal component analysis (PCA) and t‐Distributed Stochastic Neighbour Embedding (tSNE) show good separation between the two subgroups, indirectly supporting successful classification. (D) Correlation analysis between clusters and clinical charcteristics.

By analysing public data, we observed that 14 m7G‐regulated genes were significantly differentially expressed between HER2‐negative and HER2‐positive breast cancer patients (Figure 1A). To classify m7G methylation modification patterns, we performed a consensus unsupervised cluster analysis on 14 m7G regulators (showing significant differences) using the ‘Consensus Cluster Plus’ R software package. Consistent cluster analysis showed that when the cluster number was *K* = 2, patients could be divided into two groups (Figure 1B). Subgroups were termed m7G cluster 1 and cluster 2. Also, consensus clustering CDF results showed that when *K* = 2, the grouping was optimal. Furthermore, PCA and tSNE showed that m7G cluster 1 and m7G cluster 2 were well differentiated (Figure 1C). To further verify the significance of these clusters in HER2‐negative breast cancer, we examined correlations between patient survival and clinical characteristics across clusters. We observed no significant statistical differences in survival analysis (*p* = 0.084), but a certain degree of discrimination was observed, but this was potentially related to the relatively small sample size. Additionally, correlation analysis between clusters and clinical characteristics indicated a significant correlation with clinical stage and pathological type (Figure 1D).

### 3.2 Prognostic Model Construction and a Preliminary Evaluation of HER2‐Negative Breast Cancer Patients Based on m7G Regulatory Genes

**FIGURE 2:**
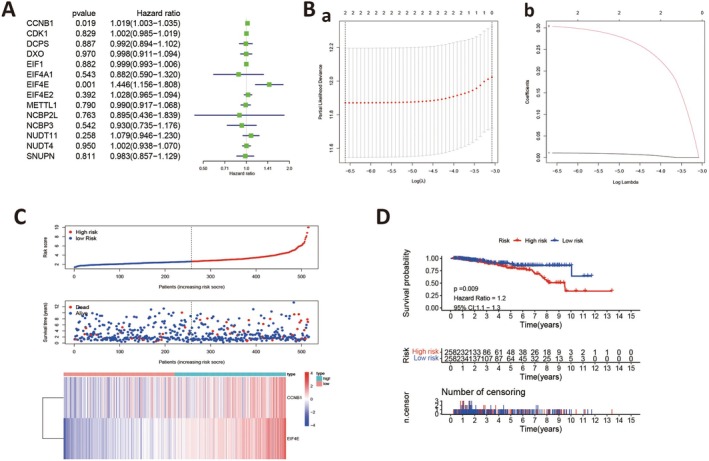
Prognostic model and preliminary evaluation of HER2‐negative breast cancer patients based on m7G regulatory genes. (A) Univariate Cox regression identified two m7G regulatory genes with prognostic value (CCNB1 and EIF4E). (B) (a, b) A prognostic model was constructed based on these two genes using LASSO regression. (C) Each patient received a risk score according to the model, with higher scores indicating a higher probability of death. A heatmap shows that CCNB1 and EIF4E are highly expressed in high‐risk patients, suggesting both genes are associated with poor prognosis. (D) Survival analysis indicates that patients with higher scores have worse survival outcomes.

To evaluate the impact of m7G‐regulated genes in HER2‐negative breast cancer, we constructed a prognostic model based on these genes. First, a primary gene screen associated with HER2‐negative expression was performed using univariate Cox regression analysis (Figure 2A). To maintain the prognostic value of our prognostic signature, a p cut‐off value was set at 0.05, and two genes (*CCNB1* and *EIF4E*) were identified (Figure 2A). Then, a LASSO Cox regression analysis screen was conducted, and a two‐gene signature was identified with an ideal λ value (Figure 2B). The risk score was computed as 0.0104873714482289 * *CCNB1 expression + 0.3036812520118* * *EIF4E* expression. By taking the median risk score as the cut‐off point, patients were divided into high‐ and low‐risk groups. Also, survival status, survival time and gene expression (both genes) were compared between groups (Figure 2C,D). From analyses, patients in the high‐risk group had worse prognostic outcomes when compared with the low‐risk group. Additionally, *CCNB1* and *EIF4E* were highly expressed in high‐risk patients, which suggested that both were potentially related to poor prognostic outcomes (Figure 2C,D).

### 3.3 Model Assessment

**FIGURE 3:**
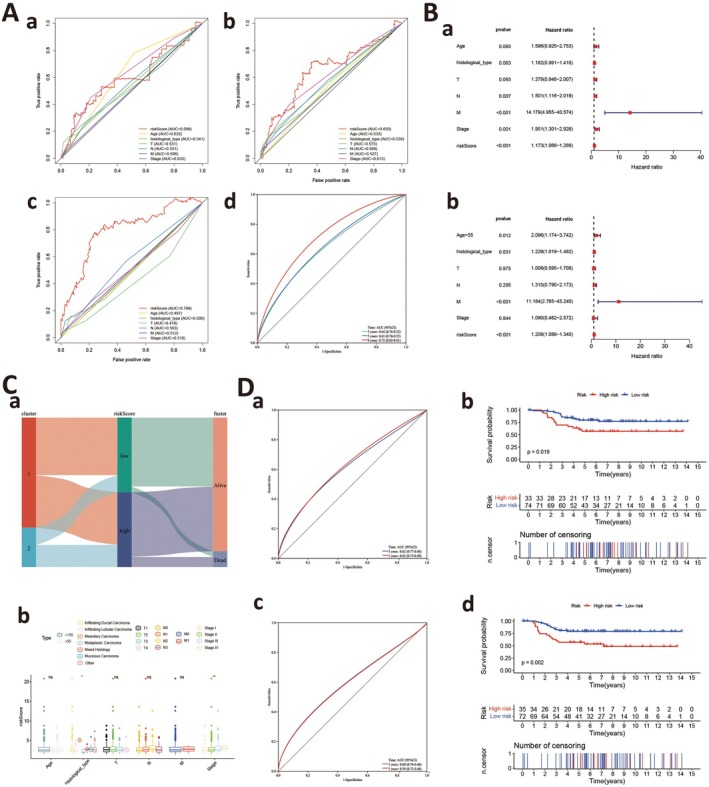
Assessment of the model. (A) (a–c) Multi‐factor ROC curves for years 3, 5 and 8; (d) risk score ROC curves for years 3, 5 and 8. (B) (a) Univariate regression evaluation of prognostic capability for each indicator; (b) multivariate regression evaluation of prognostic capability for each indicator. (C) (a) Sankey diagram showing connections between different classifications, risk groups and survival status; (b) correlation between risk score and various clinical indicators, indicating associations with stage and pathological classification. (D) (a, b) ROC and survival analysis evaluating risk score's ability to predict OS in the GEO database; (c, d) ROC and survival analysis evaluating risk score's ability to predict PFS in the GEO database.

ROC curves were used to evaluate risk model accuracy. In 3‐, 5‐ and 8‐year multivariate ROC analyses, risk scores showed a better predictive ability when compared with other clinical factors (AUC = 0.596, 0.650 and 0.796, respectively) (Figure 3A,a–c). Analyses also showed that 3‐, 5‐ and 8‐year area under the ROC curve risk score values were 0.64, 0.65 and 0.72, respectively (Figure 3A,d). Additionally, in univariate Cox regression analysis, lymph node metastasis (Hazard ratio (HR) = 1.501, *p* = 0.007), distant metastasis (HR = 14.179, *p* < 0.001), stage (HR = 1.951, *p* < 0.001) and risk scores (HR = 1.173, p < 0.001) were significantly associated with OS (Figure 3B,a). However, multivariate Cox regression analyses showed that age (HR = 2.096, *p* = 0.012), histological type (HR = 1.229, *p* = 0.031), distant metastasis (HR = 11.184, *p* < 0.001) and risk scores (HR = 1.208, *p* < 0.001) were independent risk factors for OS (Figure 3B,b). These results indicated that risk scores demonstrated good predictive ability in both ROC analyses and independent prognostic testing in evaluating outcomes.

To further explore relationships between risk scores and patient clinical characteristics, we used a Sankey diagram to identify associations between different subtypes, risk groups and survival status (Figure 3C,a). As shown (Figure 3C,b), a correlation between risk scores and various clinical indicators was identified, highlighting significant associations between risk scores and stage, and also pathological subtype. In the GEO database, we validated risk score prognostic values using ROC and survival curve analyses. In OS predictions, ROC analyses indicated that risk score AUC values, predicting 3‐ and 5‐year OS rates, were both 0.62 (95% confidence interval (CI): 0.77–0.48 and 0.75–0.49, respectively) (Figure 3D,a). Survival curve analysis showed that high‐risk group patients were associated with poorer survival outcomes (*p* = 0.019) (Figure 3D,b). In PFS predictions, ROC analyses showed that risk score AUC values, predicting 3‐ and 5‐year PFS rates, were 0.60 (95% CI: 0.74–0.46) and 0.59 (95% CI: 0.73–0.46), respectively (Figure 3D,c). Similarly, survival curve analyses also indicated that high‐risk group patients were associated with worse survival outcomes (*p* = 0.002) (Figure 3D,d).

**FIGURE 4:**
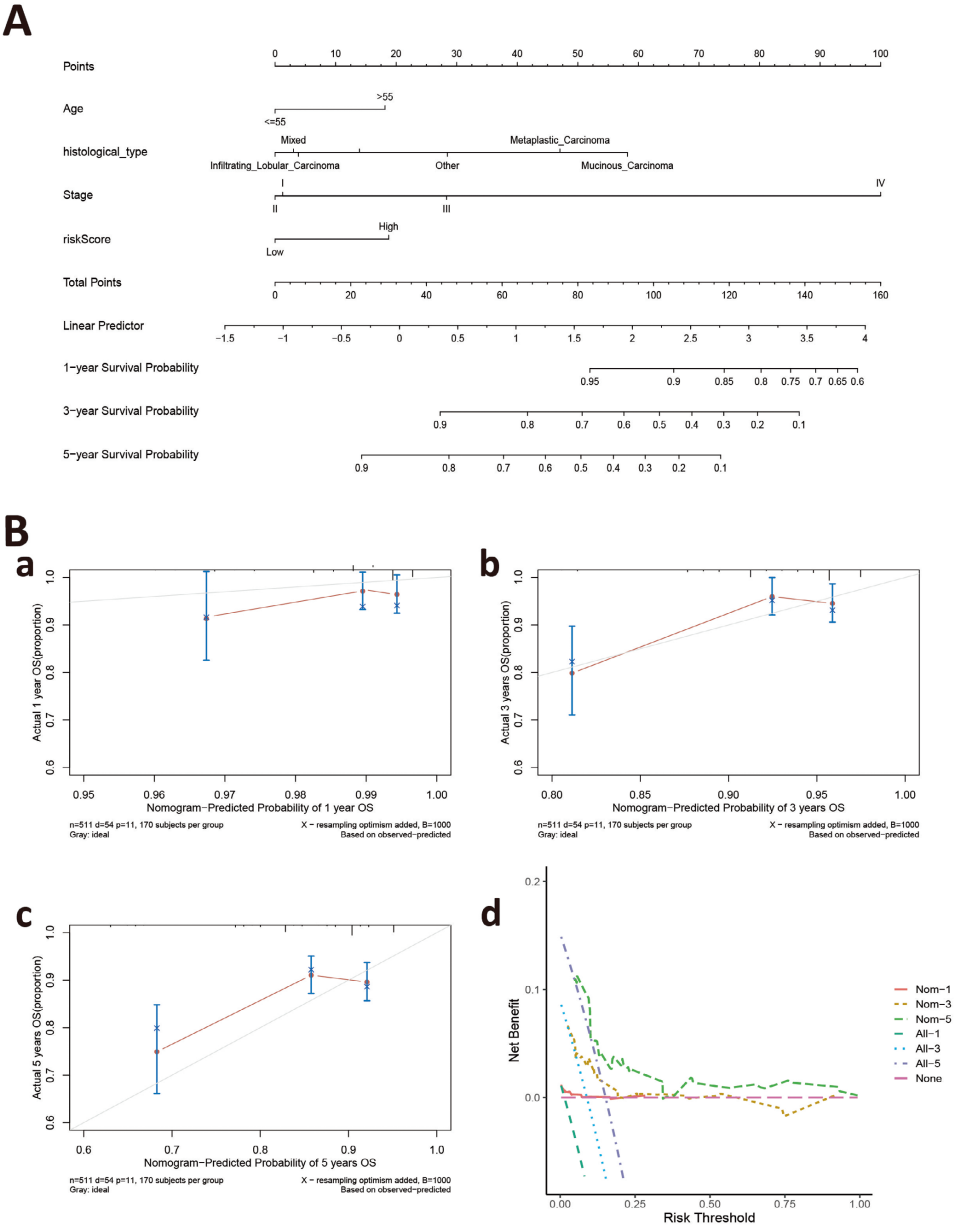
Construction of nomogram. (A) A nomogram was constructed by integrating clinical data and risk scores. (B) (a–d) Calibration and decision curve analysis (DCA) curves of the nomogram indicate its prognostic value.

Nomograms are typically used to directly calculate and display the contribution of different variables to a disease outcome, helping clinicians evaluate their importance and influence. Incorporating all parameters, we constructed a nomogram to predict 1‐, 3‐ and 5‐year OS rates in patients (Figure 4A). Furthermore, calibration and decision curve analysis plots were used to test if the nomogram was concordant with 1‐, 3‐ and 5‐year predictions (Figure 4B).

### 3.4 Functional Differentially Expressed m7G Gene Enrichment Between Groups

**FIGURE 5:**
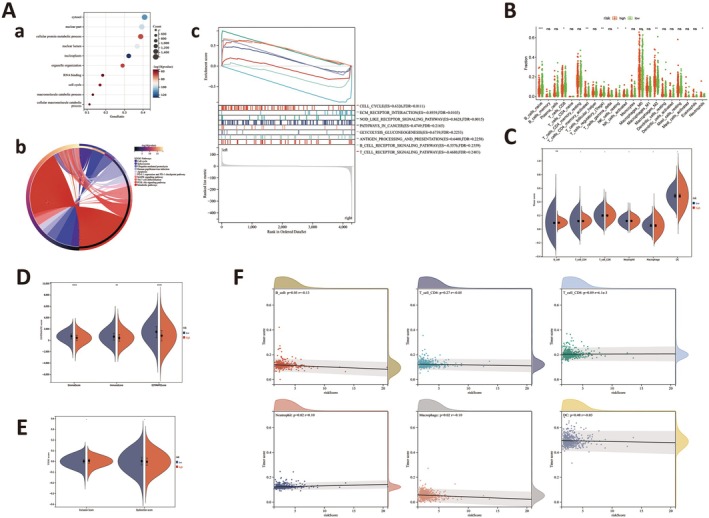
Differential pathway analysis between high‐risk and low‐risk groups. (A) (a) GO analysis; (b) KEGG analysis; (c) GSEA analysis. All three analyses enriched the cell cycle pathway, suggesting its critical role between the two groups; immune‐related pathways were also enriched in KEGG and GSEA. (B) CIBERSORT algorithm was used to calculate immune cell infiltration differences between the two groups. (C) TIMER algorithm was used to calculate immune cell infiltration differences between the two groups. (D) ESTIMATE algorithm was used to analyse immune scores between the two groups. (E) TIDE algorithm was used to analyse immunogenicity. (F) Significant correlations were observed between risk score and certain immune cells.

We divided patients into risk groups according to the aforementioned risk signatures and screened 858 differentially expressed m7G‐related genes using |log_2_ FC| > 1 and *p* < 0.05 criteria. Functional enrichment analysis also provided a biological understanding of these genes. The top 10 biological process terms from GO enrichment analyses and the top 10 KEGG pathways are shown (Figure 5A,a,b, respectively). GSEA enrichment results are also shown (Figure 5A,c). Critically, these enrichment analyses identified significant changes in cell cycle processes between groups.

### 3.5 Immunological Analyses

Intriguingly, KEGG and GSEA enrichment pathways were significantly associated with immunological functions. Therefore, we performed immunological analyses between groups. According to the CIBERSORT algorithm, naive B, CD8 T, regulatory T and activated natural killer (NK) cell infiltration was significantly higher in the low‐risk group when compared to the high‐risk group. Conversely, high‐risk patients showed higher activated CD4 memory T and resting NK cell, M2 macrophage and neutrophil infiltration levels (Figure 5B). As shown (Figure 5C), TIMER algorithm results indicated a significant difference in B cell infiltration scores between groups. Also, low‐risk group patients had significantly higher immune and ESTIMATE scores when compared to high‐risk patients (*p* < 0.05) (Figure 5D). TIDE immunogenicity scores comparing low‐ and high‐risk groups are shown (Figure 5E). A violin plot indicated no significant differences in exclusion and dysfunction scores between groups. We also examined correlations between risk and immune cell infiltration scores using TIMER (Figure 5F), which showed a significant positive correlation between risk scores and neutrophil infiltration (*r* = 0.10, *p* = 0.02) and a significant negative correlation with macrophage infiltration (*r* = −0.10, *p* = 0.02).

### 3.6 Evaluating Market Drugs

**FIGURE 6:**
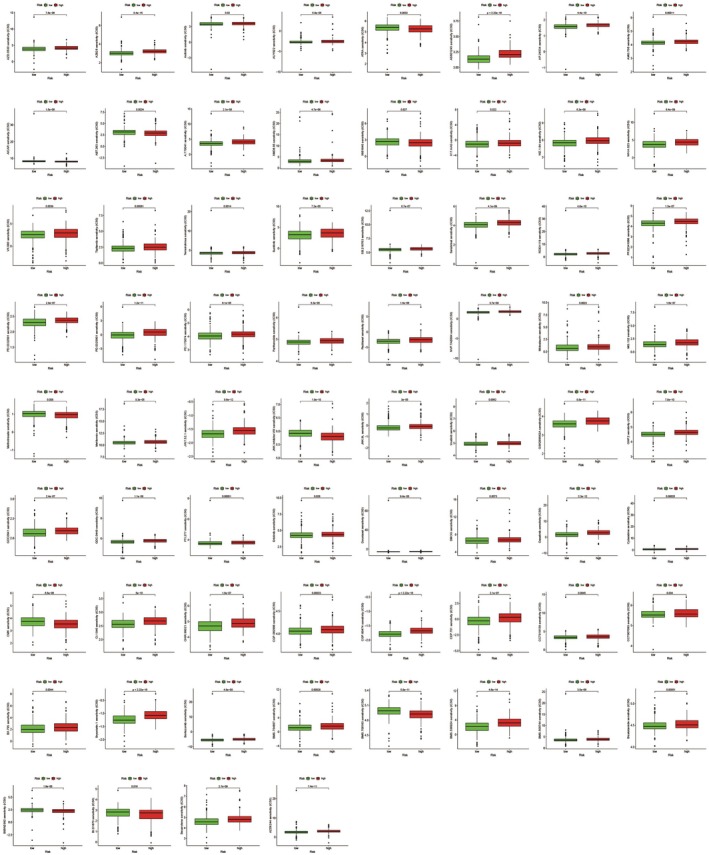
Evaluation of marketed drugs. Potential small‐molecule drugs for treating high‐ and low‐risk groups were screened using the GDSC database.

Using the GDSC database, we analysed small‐molecule IC50 values between high‐ and low‐risk groups. We identified 86 drugs, including AZD.0530, AZ628 and axitinib, which exhibited significant IC50 differences between groups (Figure 6).

### 3.7 TMB

The top 15 mutated genes in high‐ and low‐risk groups are shown (Figure S1A), including *PIK3CA, TP53, TTN, CDH1, GATA3, MUC16, TBX3, DYNC2H1, TG, KDM6A, PEG3, CBFB, CCDC66, PCDH11X and GRIK2*. Of these, *PIK3CA, CDH1, TBX3, DYNC2H1, TG, KDM6A, PEG3, CBFB, CCDC66, PCDH11X* and *GRIK2* mutation rates showed significant differences between groups. Additionally, the high‐risk group exhibited a higher TMB when compared to the low‐risk group (Figure S1B).

### 3.8 *EIF4E* Promotes HER2‐Negative Breast Cancer Cell Proliferation, Migration and Invasion

**FIGURE 7:**
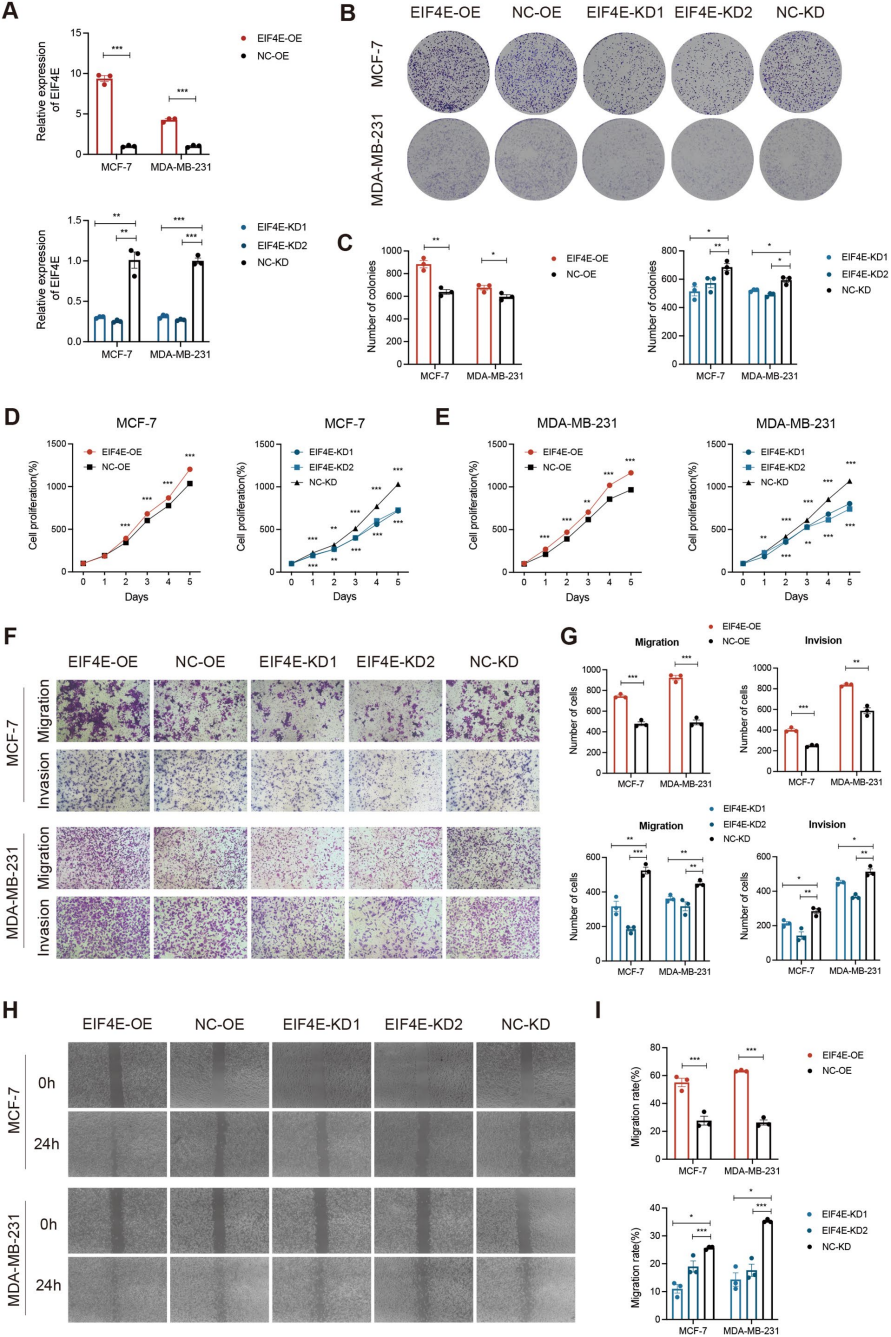
EIF4E promotes the proliferation, migration and invasion of HER2‐negative breast cancer cells. (A) qPCR was used to assess the efficiency of EIF4E overexpression and knockdown. (B, C) Colony formation assays were conducted on MCF7 and MDA‐MB‐231 cells with EIF4E overexpression or knockdown after 14 days of culture. (D, E) The CCK‐8 assay was used to evaluate the proliferation ability of MCF7 and MDA‐MB‐231 cells with EIF4E overexpression or knockdown. (F, G) Transwell assays demonstrated the role of EIF4E in cell migration and invasion. (H, I) Wound healing assays were performed at 0 and 24 h on MCF7 and MDA‐MB‐231 cells to assess the role of circMETTL3 in cell motility. *p < 0.05; **p < 0.01; ***p < 0.001.

*EIF4E* was identified as the highest risk gene in our prognostic model, prompting us to investigate its biological functions in MCF‐7 and MDA‐MB‐231 cells. *EIF4E* pcDNA and siRNA reagents were transfected into cell lines to facilitate *EIF4E* overexpression and knockdown, respectively, with qPCR confirming transfection efficiency (Figure 7A). Colony formation and CCK8 assays were performed to examine *EIF4E* overexpression and silencing effects on HER2‐negative breast cancer cell proliferation, and showed that *EIF4E* overexpression significantly enhanced colony formation and proliferation, while *EIF4E* knockdown markedly reduced levels (Figure 7B–E).

Additionally, Transwell assays were performed to assess the impact of *EIF4E* on cell migration and invasion. Migrating and invading cells were significantly higher in *EIF4E*‐overexpression (EIF4E‐OE) assays when compared with negative controls (NC), while *EIF4E* knockdown caused the opposite effects in assays (Figure 7F,G). Consistent with these findings, wound healing assays showed that upregulated *EIF4E* greatly enhanced cell motility, whereas its downregulation impaired motility (Figure 7H,I).

Therefore, *EIF4E* appeared to promote HER2‐negative breast cancer progression by enhancing cell proliferation, migration and invasion.

### 3.9 *EIF4E* Promotes Disease Progression by Regulating Wnt Signalling and Extracellular Matrix (ECM) Components

**FIGURE 8:**
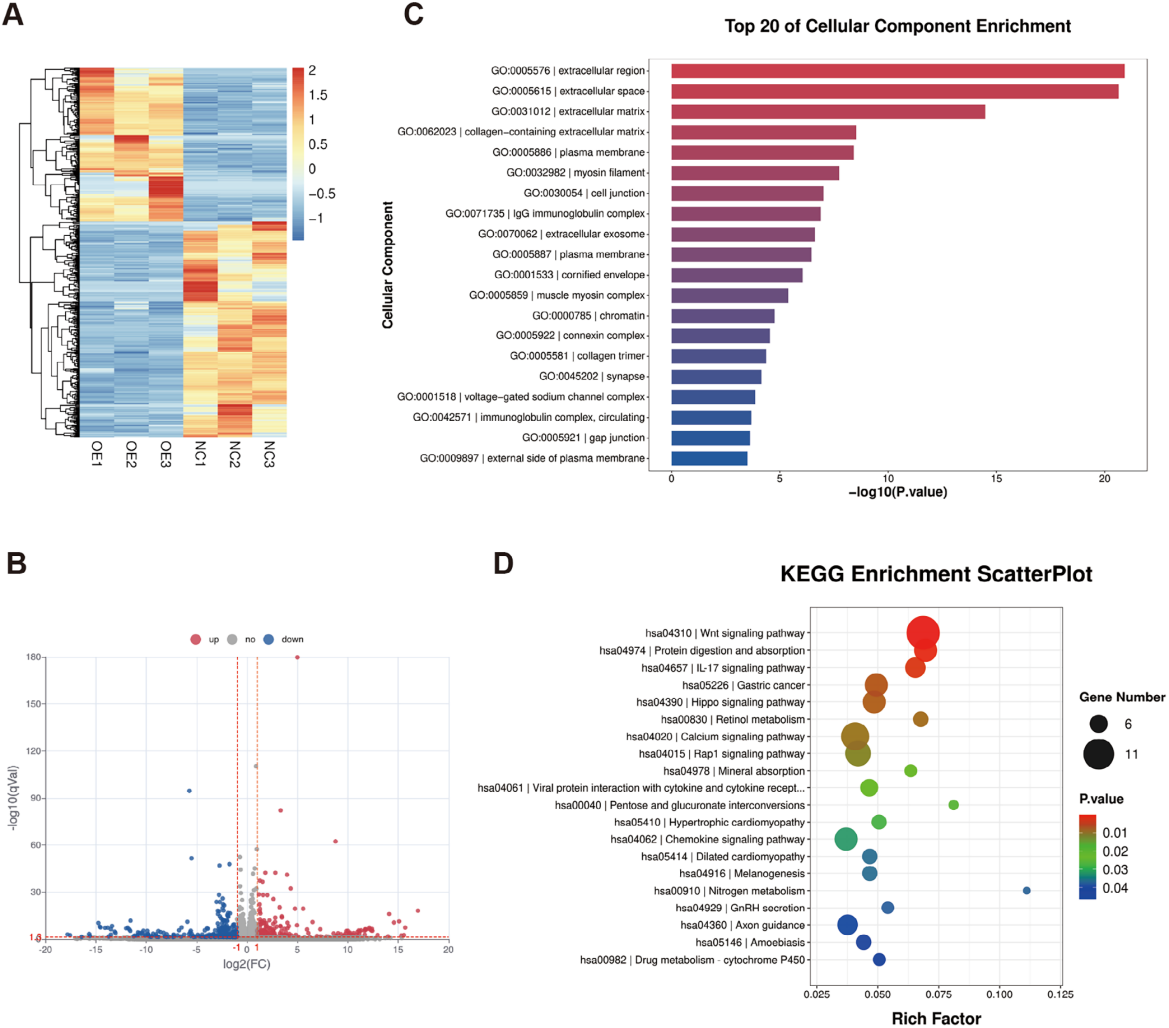
EIF4E promotes HER2‐negative breast cancer progression by regulating the Wnt signalling pathway and extracellular matrix components. (A) Heatmap showing gene expression profiles at different levels of EIF4E expression. (B) Volcano plot indicating upregulated and downregulated genes modulated by EIF4E. (C) KEGG pathway analysis of differentially expressed genes. (D) GO pathway analysis of differentially expressed genes.

To further investigate how *EIF4E* contributed to HER2‐negative breast cancer progression, in cells overexpressing *EIF4E* and controls, RNA was isolated and underwent RNA‐seq. A heatmap was then generated showing gene expression profiles under different *EIF4E* expression levels (Figure 8A). In total, 198 genes were significantly upregulated and 278 significantly downregulated (fold change > 2, *p* < 0.05) (Figure 8B). We then performed KEGG and GO pathway analyses to explore potential gene functions. KEGG analyses indicated that differentially expressed genes induced by elevated *EIF4E* levels were primarily enriched in the Wnt signalling pathway (Figure 8C). Additionally, GO analysis showed that genes were mainly enriched in extracellular components, particularly the ECM (Figure 8D).

Wnt signalling is an ancient and highly conserved signalling cascade required for animal growth and development. It is involved in many different processes, including cancer cell proliferation, stem cell regulation, apoptosis, autophagy, metabolism, inflammation, immunity, microenvironment dynamics, resistance, ion channel activity, heterogeneity, epithelial–mesenchymal transition, migration, invasion and metastasis [40, 41]. Overactivated Wnt signalling causes abnormal cellular proliferation and differentiation, ultimately resulting in tumourigenesis. In breast cancer, aberrant Wnt signalling has key roles throughout tumour initiation to metastasis [42].

The ECM is a fundamental component of tissues and organs, providing structural support and regulating cellular behaviours [43]. Recent research has highlighted a significant role for the ECM in solid tumours, with it being one of the most dynamic components in the tumour microenvironment, acting as a modulator in tumour treatment and a therapeutic target [44, 45, 46]. The ECM is also a major component of the metastatic microenvironment in breast cancer and a key driver in disease progression [47]. We hypothesise that elevated *EIF4E* levels promote HER2‐negative breast cancer cell proliferation, migration and invasion by regulating molecules associated with Wnt signalling and ECM components, thereby contributing to disease progression and poorer patient outcomes.

## 4 Discussion

In recent years, precision medicine has become increasingly influential in cancer research and treatment, particularly for complex, heterogeneous diseases like breast cancer. The concept tailors treatment plans to the molecular characteristics of a patient's tumour, thereby improving therapeutic efficacy while minimising adverse effects. This approach has been particularly effective in breast cancer, where molecular subtypes, such as hormone receptor‐positive, HER2‐positive and triple‐negative breast cancer, require unique therapeutic strategies. However, in HER2‐negative breast cancer, effective molecular targets are lacking and thus require significant research. In this study, we investigated m7G regulatory gene roles in HER2‐negative breast cancer, constructing a prognostic model to assess patient outcomes and exploring potential novel therapeutic targets.

Breast cancer encompasses a diverse group of diseases with varying molecular profiles, necessitating personalised therapeutic approaches. The application of different HER2‐targeted therapies, such as trastuzumab and pertuzumab, has substantially improved outcomes for HER2‐positive patients. Similarly, CDK4/6 inhibitors in combination with endocrine therapy have prolonged PFS in HR+/HER2‐ patients. However, options for HER2‐negative breast cancer patients are limited. Therefore, understanding and identifying novel molecular markers could improve prognostic outcomes and tailor therapy for these patients. To bridge this gap, we examined m7G‐related gene expression.

RNA modifications, particularly m6A and m7G, are crucial gene expression regulators. They influence RNA stability, splicing, translation and other processes, significantly impacting tumorigenesis and metastasis. Specifically, dysregulated m7G‐related enzymes, such as METTL1 and WDR4, are implicated in multiple cancer types. However, in breast cancer, these modifications have not been as thoroughly investigated, in particular HER2‐negative breast cancer.

In this study, we examined m7G regulatory gene expression in HER2‐negative and ‐positive breast cancer patients. By analysing 14 m7G‐regulated genes, significant differential expression was identified between patients. Furthermore, our consensus clustering analysis divided HER2‐negative patients into two subgroups, m7G cluster 1 and cluster 2. While survival analysis showed no significant statistical differences, clinical characteristics such as stage and pathological type were correlated with clusters, suggesting potential clinical relevance. These findings underscore the importance of m7G modifications and their regulatory genes as prospective biomarkers and therapeutic targets in HER2‐negative breast cancer.

Our prognostic model provided valuable insights into disease progression. Using univariate and LASSO Cox regression analyses, two genes, *CCNB1* and *EIF4E*, were identified as prognostic markers, after which we developed a risk score formula based on expression levels. The high‐risk group identified in this model demonstrated poorer prognosis outcomes when compared to the low‐risk group. This finding potentially emphasised *CCNB1* and *EIF4E* as indicators of unfavourable outcomes, warranting further tumour biology studies.

To assess model accuracy, our ROC analyses showed that risk scores had superior predictive capability when compared with other clinical factors. Both univariate and multivariate Cox regression analyses confirmed that risk scores, alongside distant metastasis, lymph node metastasis and stage, were significantly associated with OS. This result further validated the model's robustness and its potential for stratifying HER2‐negative breast cancer patients by risk.

Functional enrichment analysis of differentially expressed m7G genes between high‐ and low‐risk groups showed significantly altered biological processes and pathways, particularly related to the cell cycle. Notably, KEGG and GSEA analyses suggested strong associations with immune functions. Immunological analyses indicated that low‐risk patients exhibited higher CD8+ T, naive B, regulatory T and activated NK cell infiltration, suggesting a favourable immune environment. In contrast, high‐risk patients showed increased activated CD4 memory T, M2 macrophage and neutrophil infiltration, which potentially correlated with immunosuppression and tumour progression.

Furthermore, immune score analysis highlighted significant differences between high‐ and low‐risk groups. Low‐risk patients had higher immune and ESTIMATE scores, while neutrophil infiltration positively correlated with risk scores. These findings indicated that the immune microenvironment had a pivotal role in our prognostic model and should be explored further as a potential therapeutic avenue for HER2‐negative breast cancer.

*EIF4E* was a key gene identified in our prognostic model and underwent functional assays to characterise its role in HER2‐negative breast cancer. *EIF4E* overexpression significantly enhanced cell proliferation, migration and invasion, while silenced *EIF4E* reduced these malignant characteristics. These findings aligned with previous studies that identified *EIF4E* as an oncogenic factor in various cancers, where it promoted tumour progression by enhancing specific mRNA translation processes in cell growth and survival [48, 49, 50, 51, 52].

In the context of HER2‐negative breast cancer, *EIF4E* may be a viable therapeutic target. Its targeting could potentially disrupt these malignancy‐associated processes, thereby inhibiting tumour progression. Developing drugs to specifically target *EIF4E* or its downstream pathways could also provide new avenues for treating HER2‐negative breast cancer patients, particularly high‐risk groups as identified by our model.

We also evaluated drug sensitivity between high‐ and low‐risk groups, identifying several small‐molecule drugs with significantly different IC50 values. Drugs like AZD0530, AZ628 and Axitinib showed potential as targeted treatments, particularly in high‐risk patients. Additionally, TMB analysis showed that high‐risk patients exhibited a higher mutation burden. A high TMB has been associated with increased neoantigen formation and enhanced immune recognition, suggesting that high‐risk patients may benefit from immune checkpoint inhibitors.

Using a robust prognostic model based on m7G regulatory genes, this study represents an important step toward personalised treatment strategies for HER2‐negative breast cancer. Our findings highlight *CCNB1* and *EIF4E* as potential prognostic markers and therapeutic targets. The differential expression and functional impact of these genes suggest significant roles in tumour progression. Moreover, immune cell infiltration and drug sensitivity analyses further support the clinical relevance of our model.

While precision medicine for breast cancer has been particularly successful for HER2‐positive and HR+ subtypes, HER2‐negative breast cancer has remained challenging due to a lack of precise therapeutic targets. M7G‐related genes identified here could provide promising avenues for treatments and may lead to novel therapeutic options for HER2‐negative breast cancer patients. Future research should focus on validating these findings in larger clinical cohorts and exploring targeted therapies against m7G‐modified genes, in particular *EIF4E*, to further improve outcomes for this subset of breast cancer patients.

## Author Contributions

**Yangyang Cui:** conceptualization (equal), data curation (lead), formal analysis (lead), funding acquisition (equal), investigation (equal), methodology (equal), supervision (equal), visualization (equal), writing – original draft (lead), writing – review and editing (lead). **Yuhan Dai:** data curation (equal), formal analysis (equal), investigation (equal), methodology (equal), software (equal), supervision (equal), visualization (equal), writing – review and editing (equal). **Yiqin Xia:** data curation (equal), formal analysis (equal), investigation (equal), methodology (equal), validation (equal), visualization (equal). **Wenxin Yu:** data curation (equal), formal analysis (equal), methodology (equal), resources (equal), software (equal), supervision (equal), validation (equal). **Jiangdong Jin:** data curation (equal), methodology (equal), resources (equal), software (equal). **Shui Wang:** conceptualization (equal), data curation (equal), investigation (equal), project administration (equal), supervision (equal), visualization (equal), writing – review and editing (equal). **Hui Xie:** conceptualization (lead), funding acquisition (equal), investigation (equal), methodology (equal), project administration (lead), supervision (equal), writing – review and editing (equal).

## Conflicts of Interest

The authors declare no conflicts of interest.

## Supporting information


**FIGURE S1.** Mutation landscape between high‐risk and low‐risk groups. (A) Waterfall plot of the top 15 mutated genes in each group. (B) Comparison of tumour mutation burden (TMB) between the two groups.

## Data Availability

The data supporting the conclusions of this article will be made available by the authors, without undue reservation.
