# Supplementary figures and images for: N7‐Methylguanine‐Related Gene Signature Highlights EIF4E as a Novel Therapeutic Target in HER2‐Negative Breast Cancer
Source: J Cell Mol Med. 2025 Aug 21;29(16):e70808. doi: 10.1111/jcmm.70808 (PMC12370542; doi:10.1111/jcmm.70808)

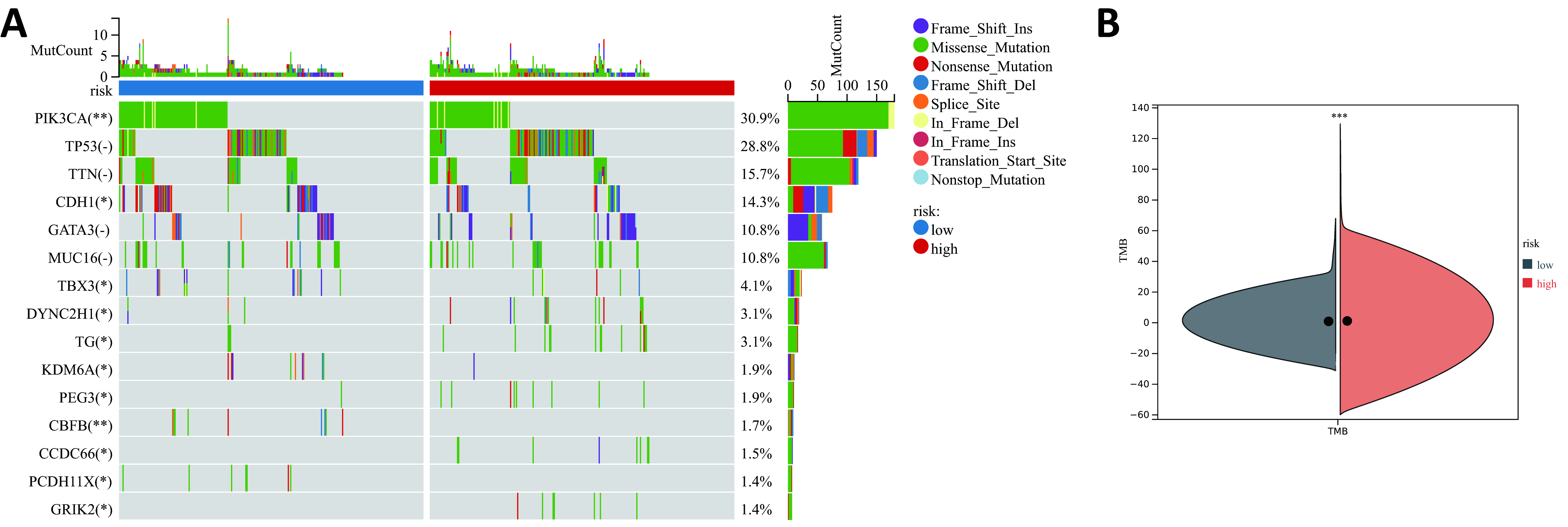

Supplement: Supplementary file 1 — FIGURE S1. Mutation landscape between high‐risk and low‐risk groups. (A) Waterfall plot of the top 15 mutated genes in each group. (B) Comparison of tumour mutation burden (TMB) between the two groups. [file JCMM-29-e70808-s001.tif]
